# Botanical Drug Puerarin Ameliorates Liposaccharide-Induced Depressive Behaviors in Mice via Inhibiting RagA/mTOR/p70S6K Pathways

**DOI:** 10.1155/2021/7716201

**Published:** 2021-10-18

**Authors:** Jia Zhao, Yizhen Jia, Wei Zhao, Huixin Chen, Xiuying Zhang, Fung Yin Ngo, Dan Luo, Youqiang Song, Lixing Lao, Jianhui Rong

**Affiliations:** ^1^Department of Chinese Medicine, The University of Hong Kong Shenzhen Hospital, Shenzhen, China; ^2^School of Chinese Medicine, The University of Hong Kong, 10 Sassoon Road, Pokfulam, Hong Kong, China; ^3^Zhu Nansun's Workstation, School of Chinese Medicine, The University of Hong Kong, 10 Sassoon Road, Pokfulam, Hong Kong, China; ^4^Yu Jin's Workstation, School of Chinese Medicine, The University of Hong Kong, 10 Sassoon Road, Pokfulam, Hong Kong, China; ^5^School of Biomedical Science, The University of Hong Kong, 21 Sassoon Road, Pokfulam, Hong Kong, China; ^6^The University of Hong Kong Shenzhen Institute of Research and Innovation (HKU-SIRI), Shenzhen, China

## Abstract

**Background:**

The depressive symptom hallmarks the progression of the neurodegenerative diseases, especially Alzheimer's disease. Bacterial infection is related to inflammation and depression. The present project thereby examined whether botanical drug puerarin could attenuate liposaccharide- (LPS-) induced depressive behaviors in mice.

**Methods:**

Adult male C57BL/6N mice were sequentially treated with LPS and puerarin and evaluated for the depressive behaviors by tail suspension test and forced swim test. The brain tissues were profiled for the molecular targets of puerarin by next-generation RNA sequencing technique. Candidate targets were further verified in LPS-treated mice, neural stem cells, and highly differentiated PC12 cell line.

**Results:**

Puerarin ameliorated LPS-induced depression in the mice. RNA sequencing profiles revealed that puerarin altered the expression of 16 genes while markedly downregulated Ras-related GTP-binding protein A (RagA) in LPS-treated mice. The effect of puerarin on RagA expression was confirmed by immunostaining, Western blot, and quantitative real-time PCR (qRT-PCR). Biochemical studies showed that puerarin inhibited RagA/mTOR/p70S6K pathway, attenuated the accumulation of mTORC1 in close proximity to lysosome, and reduced the production of proinflammatory cytokines.

**Conclusions:**

Botanical drug puerarin attenuated inflammation and depressive behaviors in LPS-challenged mice by inhibiting RagA/mTOR/p70S6K pathways. Puerarin may be a lead compound for the new antidepressant drugs.

## 1. Introduction

Alzheimer's disease (AD) is characterized by memory loss and cognitive deficit due to progressive neurodegeneration [1]. Most of AD patients suffer from depression while severe depression affects over 264 million people (https://www.who.int/). Infection and social stress dramatically increase the prevalence of depression worldwide [2]. The etiology of the depression remains elusive. Among several risk factors, neuroinflammation is recently identified to be highly related to depression [3]. The proinflammatory cytokines including interleukin- (IL-) 1*β* and IL-6 were increased whereas the anti-inflammatory cytokines including IL-10 and IL-4 were decreased in depression [4]. Moreover, bacterial lipopolysaccharide (LPS) induces inflammation and depression in mice [5–7]. Our previous study showed that LPS induced depression in mice by upregulating small guanosine triphosphatases (GTPases) RagA expression and subsequently activating mammalian target of rapamycin (mTOR)/p70S6K pathway [8]. Therefore, the RagA/mTOR/p70S6K pathway may be a therapeutic target for the treatment of depression.

The mTOR pathway is a key mechanism in the regulation of protein synthesis, cellular metabolism, and autophagy while mTOR is also implicated in the proliferation of neural stem cells and circadian rhythms [9]. Two different mTOR complexes (e.g., mTORC1 and mTORC2) execute the biological functions in response to specific stimuli [10]. The complex mTORC1 facilitates the cell growth by inhibiting autophagy and the expression of lysosomal genes whereas mTORC2 phosphorylates and activates PKB/Akt and PKC kinase, thereby promoting cell survival [11]. The mTORC1 pathway is dysregulated in the neurodegenerative diseases [12]. Nutrients promote mTORC1 translocation to the lysosomal membrane and activate the mTORC1 pathway via Rag GTPases [13]. On the other hand, Rag GTPases exist in four isoforms, namely, RagA, RagB, RagC, and RagD, and often form heterodimers (e.g., RagA/B, RagC/D). Upon stimulation by the nutrients, the complex RagA/B binds guanosine triphosphate (GTP) whereas the complex RagC/D binds guanosine diphosphate (GDP). The activation of Rag GTPases promotes the lysosomal localization of mTORC1 to facilitate the interaction of mTORC1 with the kinase activator Rheb [14, 15]. The active mTORC1 colocalizes with the lysosomal biomarker lysosome-associated membrane protein (LAMP2) and regulates the lysosomal biogenesis [16, 17]. Moreover, the activation of mTORC1 induces the phosphorylation of the downstream targets including p70 S6 kinase (p70S6K) and eukaryotic translation initiation factor 4E-binding protein 1 (4E-BP1), thereby driving the onset of depression [18, 19].

**Figure 1:**
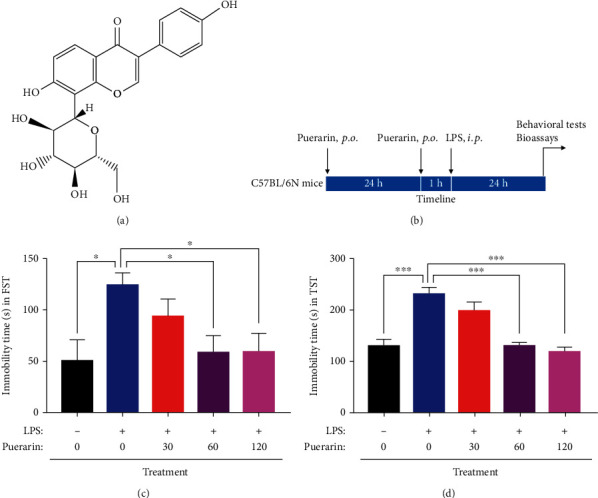
Puerarin ameliorated depressive-like behaviors in the LPS-challenged mice. (a) The chemical structure of puerarin was shown. (b) The design of the animal experiments. Mice were separated into 5 groups: control, LPS, and LPS+puerarin (30, 60, or 120 mg/kg). After drug treatment, mice were assessed for depressive-like behaviours, and brain tissue was collected for the bioassays. (c) Forced swim test (FST). The depressive-like behaviors of mice were evaluated by measuring immobility time during 4 min. (d) Tail suspension test (TST). The depressive-like behaviors of mice were evaluated by measuring immobility time during 6 min. The data were presented as means ± SEM (*n* = 6). ^∗^*p* < 0.05; ^∗∗^*p* < 0.01; ^∗∗∗^*p* < 0.001.

The conventional antidepressant drugs have been found to have various side effects in the past couple of decades [20]. As an example, ketamine exhibits rapid antidepressant effects via regulating 4E-BPs [19]. However, ketamine causes various side effects and even toxicity because its abuse harms people and society [21]. On the other hand, natural products could effectively attenuate LPS-induced depression in mice [22]. Along this line, our group recently demonstrated that C-glucosylated isoflavone puerarin (Figure 1(a)), as the main active compound from the herbal medicine Radix puerariae, attenuated pain and depression in mice with spared nerve injury [23]. Consistently, other studies also showed that puerarin attenuated depressive-like behaviors by upregulating FGF_2_ expression or via estrogen and BDNF signaling pathways [24, 25]. Thus, it would be of importance to further dissect the mechanism by which puerarin exhibits antidepressant activity.

In the present study, we hypothesized that puerarin could attenuate the depressive-like behaviors in LPS-challenged mice. Following puerarin treatment, depressive-like behaviors were assessed in the LPS-challenged mice. The brain tissues were subjected to the transcriptome profiling for the molecular targets of puerarin. The candidate targets of puerarin were further verified in LPS-treated mice, neural stem cells, and highly differentiated PC12 cell line.

## 2. Materials and Methods

### 2.1. Antibodies and Biochemical Reagents

The antibodies against RagA, phospho-mTOR, mTOR, phospho-p70S6K, p70S6K, and GAPDH were obtained from Cell Signaling Technology (Boston, MA, USA). HRP-conjugated anti-rabbit IgG was purchased from Sigma–Aldrich (St. Louis, MO, USA). NeuN and LAMP2 antibodies were purchased from Abcam (Cambridge, MA, USA). Alexa Fluor 488-conjugated goat anti-mouse IgG and Alexa Fluor 594-conjugated goat anti-rabbit IgG were obtained from Invitrogen (Carlsbad, CA, USA). ChamQ SYBR qPCR master mix was obtained from Vazyme (Nanjing, China). QuantiNova SYBR green PCR kit was obtained from QIAGEN (Valencia, CA, USA). Dulbecco's modified Eagle's medium (DMEM), DMEM/F12, fetal bovine serum (FBS), horse serum (HS), N2, B27, penicillin, streptomycin, basic fibroblast growth factor (bFGF), and epidermal growth factor (EGF) were obtained from Thermo Fisher Scientific (Waltham, MA, USA). The mammalian expression vector pcDNA3.1 was obtained from Invitrogen (Carlsbad, CA, USA). Puerarin was obtained from Yick-Vic Chemicals & Pharmaceuticals (Hong Kong, China). LPS, Accumax solution, RIPA buffer, and other reagents were obtained from Sigma–Aldrich (St. Louis, MO, USA) unless otherwise indicated.

### 2.2. Animals and Drug Administration

Animal experiments were approved by the Committee on the Use of Live Animals in Teaching and Research of the University of Hong Kong (CULATR 4284-17). Male C57BL/6N mice (7-8 weeks, 18–25 g) were provided by the Centre for Comparative Medicine Research, the University of Hong Kong. Animals were housed on a 12 h light–dark cycle and allowed to freely access food and water at the animal facility, the University of Hong Kong. For the behavioral experiments, puerarin was dissolved in saline containing 50% 1,2-propylene glycol. LPS was dissolved in the saline. Mice were randomly separated into 5 groups (*n* = 5): control, LPS, LPS+puerarin (30 mg/kg), LPS+puerarin (60 mg/kg), and LPS+puerarin (120 mg/kg). As outlined in Figure 1(b), following intragastric administration of puerarin for 24 h, mice sequentially received oral gavage of puerarin and intraperitoneal injection of LPS (0.083 mg/kg) with 1 h interval for another 24 h, and the mice in the control group were given the same volume of saline with or without 1,2-propylene glycol. The behavioral tests were performed after LPS treatment.

### 2.3. Behavioral Tests

Following drug treatment, mice were assessed for depressive behaviors by tail suspension test (TST) and forced swim test (FST) as previously described [23, 26].

For TST, adult mice were immobilized to the suspension bar by attaching the tail with adhesive tape. The distance of 20-25 cm was kept between apparatus floor and mouse nose. During the test over 6 min, a blinded observer recorded the immobility time of the mice.

For FST, adult mice were transferred into a plastic cylinder (30 cm high, 20 cm diameter) filling water at 22 ± 2°C to the height of 15 cm. Following 2 min habituation sessions, the immobility time was recorded by a blinded observer during the last 4 min.

### 2.4. Transcriptome Profiling by Next-Generation RNA Sequencing

Following the behavioral tests, the 3 mm frontal cortex was collected from the mouse brains (*n* = 3). The RNAs were extracted with TRIzol reagent from Thermo Fisher Scientific (Waltham, MA, USA) and purified with RNeasy plus mini kit from QIAGEN (Valencia, CA, USA). Following reverse transcription, the cDNA libraries were sequenced on the Illumina HiSeq 4000 System in BGI (Shenzhen, China). The bioinformatic analyses were performed essentially as previously described [27, 28]. Briefly, the sequence reads were mapped to the mouse grcm38_snp_tran reference sequence with HTSAT2 [29]. Ballgown was applied for the differential gene expressions [30]. The differential genes were selected based on *p* value of <0.05 and fold change (FC) of ≥2. *R* was used to make the heatmap. The network of the differential genes was established by Cytoscape (https://cytoscape.org/). The differential genes were analyzed with the Kyoto Encyclopedia of Genes and Genomes (KEGG) pathway and the enriched Gene Ontology (GO) by DAVID 6.8 (http://david.ncifcrf.gov). The *p* values less than 0.05 indicated that the differential genes were significantly enriched [31].

### 2.5. Molecular Cloning

RagA cDNA was prepared by polymerase chain reaction (PCR) amplification by using the forward primer (5′-GTACAAGCTTCAGGTGATGCCCAATACA-3′) and the reverse primer (5′-GTACGATATCGGCATTATTTCAACGCATGA-3′). PCR amplification was conducted in 50 *μ*l reaction mixture containing 10 *μ*l of 5X HF buffer, 2 *μ*l of 50 mM MgCl2, 1 *μ*l of 25 mM dNTPs mix, 2.5 *μ*l each of 20 *μ*M reverse and forward primers, 100 ng of template DNA, and 0.5 *μ*l of Phusion DNA polymerase (5 U/*μ*l, NEB, USA) in a programmable Eppendorf Thermocycler (Hong Kong, China). PCR reaction was programmed as follows: an initial denaturation of 30 seconds at 98°C, 35 cycles of 98°C for 10 seconds, 57°C for 30 seconds, and 72°C for 1 min, with a final extension at 72°C for 10 min. PCR product was purified from gel by the gel extraction kit (QIAGEN, USA). After the cleavage with HindIII and EcoRV, RagA PCR product was purified and ligated into mammalian expression vector pcDNA 3.1(+) from Invitrogen (Carlsbad, CA, USA). Following transformation and selection, pcDNA 3.1-RagA plasmid DNA was prepared in DH5*α E. coli* cells and purified with QIAGEN Plasmid Miniprep kit (Valencia, CA, USA). The clones were sequenced through Sanger DNA Sequencing Facility at BGI, Shenzhen, China.

### 2.6. Neural Stem Cell (NSC) Culture

Cortical NSCs were produced from C57BL/6N mouse embryos on embryonic day 14 as previously described [32, 33]. The NSCs were plated in a 90 mm dish at the density of 1 × 10^5^ cells/ml and cultured in DMEM/F12 medium supplemented with 2% B27, 1% N2, 20 ng/ml bFGF, 20 ng/ml EGF, and 1% streptomycin and penicillin at 37°C for 5-7 days. The single cell suspension of NSCs was seeded in the 6-well plates coated with poly-D-lysine for 24 h and treated with 200 ng/ml LPS with or without puerarin at the doses of 10, 25, and 50 *μ*M for 24 h.

### 2.7. Highly Differentiated PC12 Cell Culture and Transfection

Highly differentiated PC12 cell line was obtained from National Collection of Authenticated Cell Cultures (Shanghai, China). The cells were cultured in DMEM supplemented with 10% HS, 5% FBS, 1% penicillin and streptomycin at 37°C, and 5% CO_2_. For the transfection, the cells were seeded in the 6-well plates at the density of 1.5 × 10^5^ cells/ml for 24 h. The pcDNA3.1(+) vector or pcDNA3.1(+)-RagA plasmid was transfected into the cells by using Effectene Transfection Reagent from QIAGEN (Valencia, CA, USA) for 24 h.

### 2.8. Western Blot Analysis

The frontal cortex tissues or primary mouse neural stem cells were lysed in RIPA buffer for 30 min. The cellular proteins (30 *μ*g) or brain tissue proteins (70 *μ*g) were separated by 10% SDS-polyacrylamide gels. The PVDF membranes were blocked in the 5% BSA in Tris-buffered saline and 0.1% Tween-20 buffer overnight at 4°C. Blots were incubated with primary antibodies overnight and secondary antibodies for 3 h. Subsequently, blots were detected with Amersham™ ECL™ Select Western blotting detection reagents from GE Healthcare (Uppsala, Sweden) [34].

### 2.9. Immunostaining

After the behavioral tests, a mixture solution of ketamine/xylazine was used for the mice anesthesia, and 4% paraformaldehyde (PFA) in 0.01 M phosphate-buffered saline (PBS) was used for the perfusion. 4% PFA was used to fix the mouse brains overnight at 4°C. The thickness of the coronal sections of the 3 mm frontal cortex was 40 *μ*m. NeuN and RagA primary antibodies were used to probe the tissues overnight at 4°C. The tissues were then incubated with secondary antibodies including Alexa Fluor 488-conjugated goat anti-mouse IgG and Alexa Fluor 594-conjugated goat anti-rabbit IgG for 2 h at room temperature (RT). 4′-6-Diamidino-2-phenylindole (DAPI) was used to stain the cell nucleus. The slides were examined under a Zeiss LSM 700 confocal microscopy (Jena, Germany). The images were analysed by NIH ImageJ software (http://imagej.net/ImageJ2) [23].

For cell culture, the NSCs were treated with 200 ng/ml LPS with or without puerarin at the doses of 10, 25, and 50 *μ*M for 24 h and fixed with 4% PFA. Blocking was performed in 5% normal goat serum for 2 h at RT. The NSCs were sequentially incubated with primary antibodies against mTOR and LAMP2 (FITC) overnight at 4°C and Alexa Fluor 594 goat anti-rabbit IgG secondary antibody for 2 h at RT. DAPI was used to stain the cell nucleus. The slides were examined under a Carl Zeiss fluorescence microscopy (Jena, Germany) [8].

### 2.10. Quantitative Real-Time PCR (qRT-PCR)

The mouse frontal cortex tissues and highly differentiated PC12 cells were analysed for gene expression by qRT-PCR technique as previously described [35]. Briefly, total RNAs were extracted from the frontal cortex tissues and highly differentiated PC12 cells with TRIzol reagent from Thermo Fisher Scientific (Waltham, MA, USA). The cDNAs were prepared from total RNAs with RevertAid RT Reverse Transcription kit from Thermo Fisher Scientific (Waltham, MA, USA). PCR detection was performed with a SYBR Green PCR kit from QIAGEN (Valencia, CA, USA) and Vazyme (Nanjing, China). The primers for various mouse genes were obtained from QIAGEN as follows: RagA (Mm_Rraga_1_SG; QT00257880), FZD1 (Mm_Fzd1_1_SG; QT00290542), Fitm2 (Mm_Fitm2_1_SG; QT00147903), Camkk2 (Mm_Camkk2_1_SG; QT00162449), Ap3S2 (Mm_Ap3s2_1_SG; QT00148428), Mesdc1(Mm_Mesdc1_1_SG; QT00292187), GAPDH (Mm_Gapdh_3_SG; QT01658692), and *β*-actin (Mm_Actb_1_SG; QT 00095242). The primers for rat genes were as follows: RagA (NM_053973), F: 5′-GGAACCTGGTGCTGAACCTGTG-3′, R: 5′-GGATGGCTTCCAGACACGACTG-3′; IL-6 (NM_012589), F: 5′-GCCCACCAGGAACGAAAGTCAA-3′, R: 5′-GGAAGGCAGTGGCTGTCAACAA-3′; IL-1*β* (NM_031512), F: 5′-AATGCCTCGTGCTGTCTGACC-3′, R: 5′-GTGGGTGTGCCGTCTTTCATCA-3′; *β*-actin (NM_031144), F: 5′-GTATGCCTCTGGTCGTACCA-3′, R: 5-CTCTCAGCTGTGGTGGTGAA-3′. The data were normalized to *β*-actin or GAPDH. The relative expression level of genes was indicated by 2 − ∆∆Ct.

### 2.11. Statistical Analysis

The data were presented as mean ± SEM for body weight, behavioral experiments, and qRT-PCR in the animal experiments and mean ± SD for other experiments. The comparisons of multiple groups were performed using one-way analysis of variance (ANOVA) followed by Dunnett's test. GraphPad Prism 7 (La Jolla, CA, USA) was used to analyze the data. The *p* values of less than 0.05 were regarded as statistically significant.

## 3. Results

### 3.1. Puerarin Attenuated the Depressive-like Behaviors in LPS-Challenged Mice

FST and TST were used to assess the effect of puerarin on the depressive behaviors in LPS-challenged mice as previously described [23]. As outlined in Figure 1(b), C57BL/6N mice were administered with LPS or in combination with puerarin. The results of FST and TST were shown in Figures 1(c) and 1(d), respectively. Indeed, compared with the control group, LPS markedly prolonged the immobility time of mice in the FST and TST. Puerarin (60 and 120 mg/kg) significantly reduced the immobility time in LPS-treated mice in FST and TST compared with LPS alone group while such drug (30 mg/kg) did not exhibit evident activities.

### 3.2. Puerarin Affected RagA and Other 15 Genes in LPS-Treated Mice

**Figure 2:**
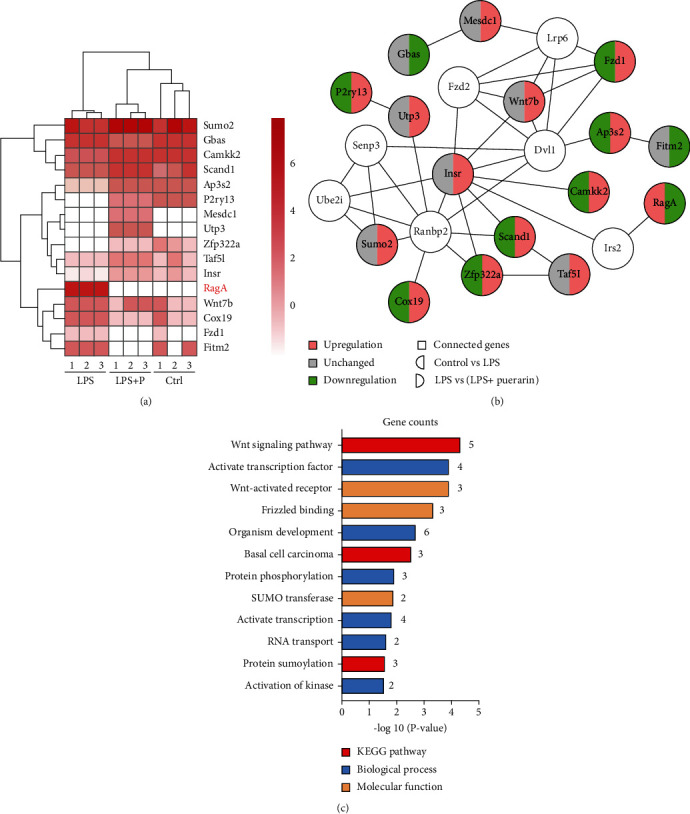
Puerarin significantly affected 16 differentiated genes in LPS-challenged mice. (a) Heatmap of differentially expressed genes. The frontal cortex tissues were collected from 3 groups of mice (i.e., control, LPS, and LPS+puerarin (60 mg/kg), *n* = 3) and profiled by next-generation RNA sequencing technology. The differentially expressed genes were identified by RNA sequencing and bioinformatics analysis. The heatmap of 16 differentially expressed genes was visualized with R language. (b) Interaction network of differentially expressed genes. The interaction network of 16 differentially expressed genes and 7 unchanged genes was generated by Cytoscape. The downregulated genes were described as green whereas the upregulated genes were described as red. (c) GO and KEGG pathway analysis of differentially expressed genes. Sixteen differentially expressed genes were analysed for GO and KEGG pathway by DAVID 6.8.

To discover the antidepressant mechanisms for puerarin, the mouse frontal cortex tissues were collected from control, LPS, and LPS+P groups and profiled by next-generation RNA sequencing technology. As shown in Figures 2(a) and 2(b), a total of 16 differentiated genes with *p* < 0.05 and FC ≥ 2 were identified based on the comparison groups: LPS vs. control and LPS+P vs. LPS. In particular, 10 genes were upregulated while 6 genes were downregulated in the comparison of LPS+P group vs. LPS group. Puerarin downregulated the level of RagA expression to the largest extent (FC = 0.02) based on RagA expression in LPS+P group and LPS group. The interaction network was created by including 16 differentiated genes and 7 closely related genes although no significant change was detected. The selected genes were enriched by GO and KEGG pathways. As shown in Figure 2(c), activate transcription factor (GO:0051091), Wnt-activated receptor (GO:0042813), and frizzled binding (GO:0005109) were the most significantly enriched in the GO terms “biological process” and “molecular function.” Wnt-signaling pathway was the most significantly enriched KEGG pathway (KEGG: mmu04310).

### 3.3. Puerarin Effectively Downregulated RagA in LPS-Challenged Mice

**Figure 3:**
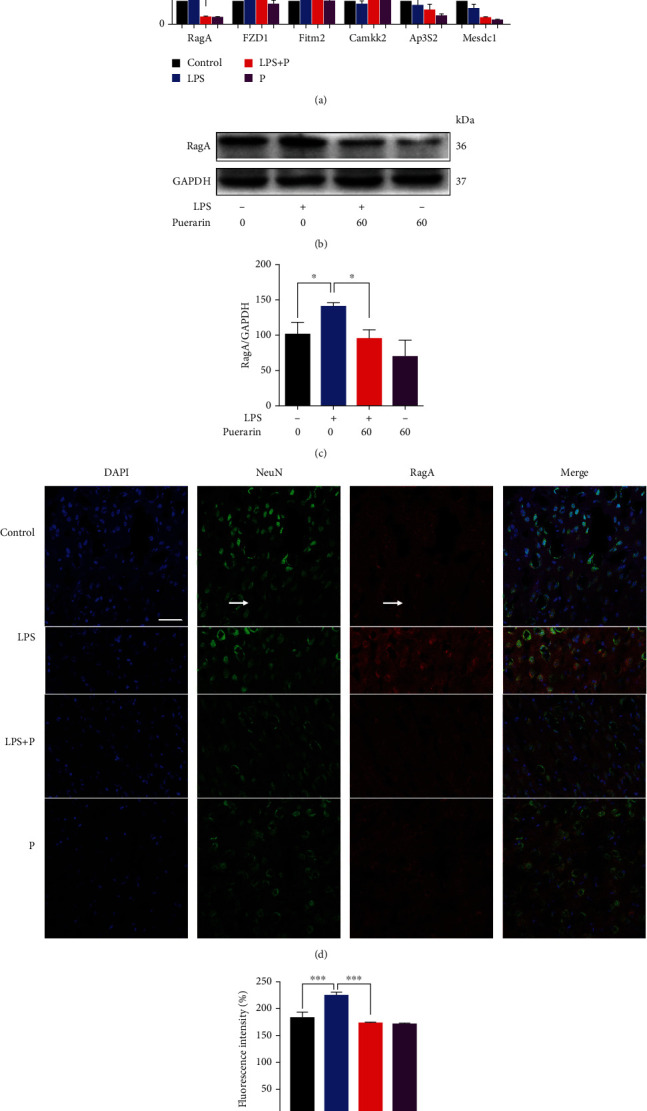
Puerarin downregulated RagA expression in LPS-challenged mice. (a) qRT-PCR quantification of 6 representative differentially expressed genes. The frontal cortex tissues were collected from 4 groups of mice (i.e., control, LPS, LPS+puerarin (60 mg/kg), and puerarin (60 mg/kg), *n* = 3) and analysed by qPCR for selected genes. The data were presented as means ± SEM (*n* = 3). ^∗^*p* < 0.05. (b) Western blot analysis of the protein expression of RagA. The frontal cortex tissues were collected from the experimental mice (*n* = 3) for Western blot analysis of RagA. (c) Quantification of RagA expression. The blots were detected by a densitometric method (*n* = 3). ^∗^*p* < 0.05. (d) Immunofluorescence detection of RagA expression in the frontal cortex. The frontal cortex tissues were collected from the experimental mice (*n* = 3) for immunohistochemical analysis for RagA expression, whereas DAPI was used to stain nuclear. NeuN was detected as the biomarker for the mature neurons. The images were captured with a Carl Zeiss 700 confocal fluorescence microscope (Jena, Germany). Scale bar, 50 *μ*m. (e) Quantification of RagA expression. Fluorescence intensity of RagA in (d) was evaluated through the densitometric method (*n* = 3). ^∗∗∗^*p* < 0.001.

To verify the results of RNA sequencing, six representative genes (i.e., RagA, FZD1, Fitm2, Camkk2, Ap3S2, and Mesdc1) were validated by qPCR technique. As shown in Figure 3(a), the brain tissues from LPS group resulted in higher mRNA levels of RagA, FZD1, and Fitm2. Puerarin (60 mg/kg) significantly decreased the mRNA levels of these genes against LPS stimulation. RagA was further verified by Western blotting and immunostaining. As shown in Figures 3(b) and 3(c), LPS significantly enhanced the protein levels of RagA whereas puerarin (60 mg/kg) significantly reduced RagA expression in LPS-treated mice. Puerarin group showed the lower level of RagA expression compared with control group. As shown in Figures 3(d) and 3(e), LPS significantly enhanced the immunofluorescence staining of RagA expression whereas puerarin (60 mg/kg) significantly decreased the immunofluorescence intensity for RagA staining against LPS stimulation. Moreover, after LPS treatment, RagA and NeuN appeared to be colocalized in the frontal cortex of mice.

### 3.4. Puerarin Inhibited the Activation of RagA/mTOR/p70S6K Pathway and the Lysosomal Translocation of mTORC1 in Neural Stem Cells

**Figure 4:**
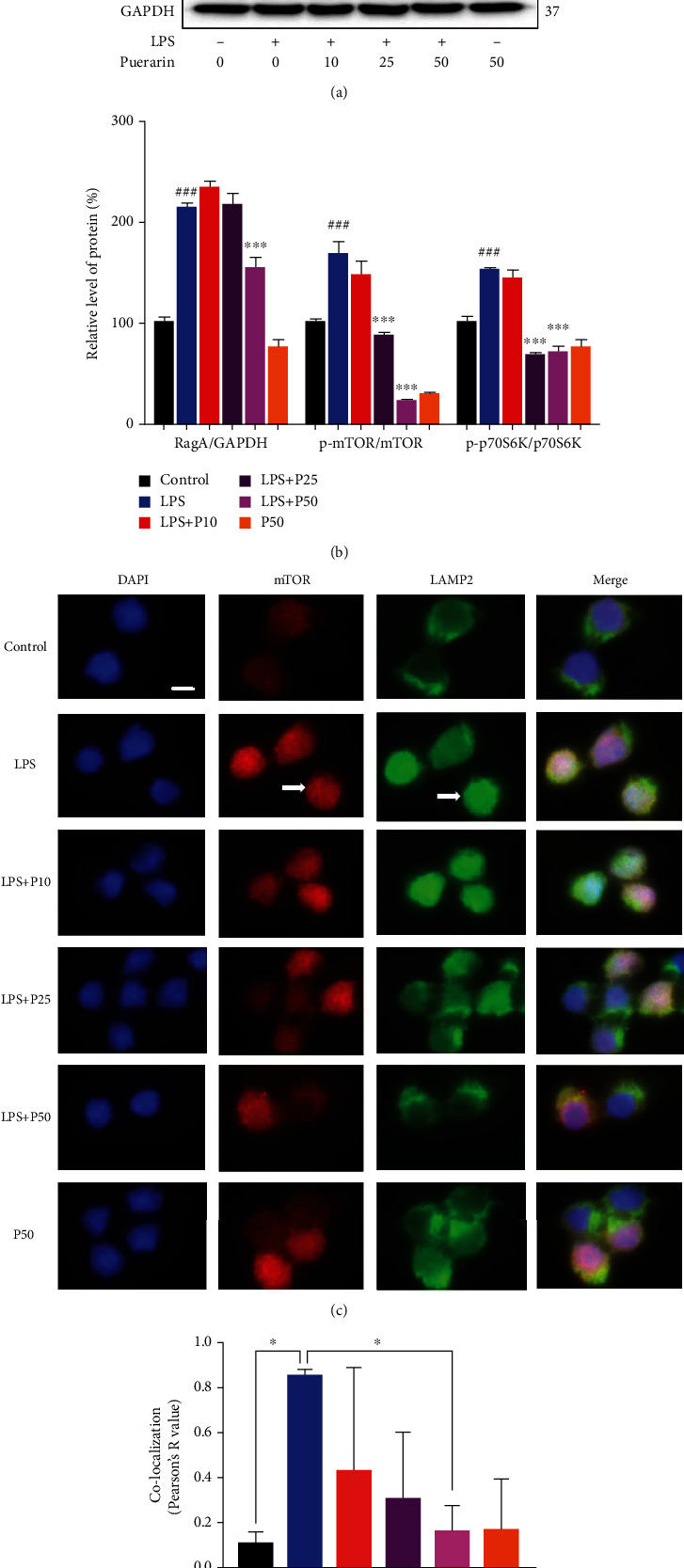
Puerarin inhibited the RagA/mTOR/p70S6K pathway and the lysosomal localization of mTORC1 in the NSCs. (a) Detection of RagA, mTOR, and p70S6K. The protein expression of RagA, mTOR, and p70S6K were analyzed by Western blot in LPS-challenged neural stem cells. After 24 h treatment with 200 ng/ml LPS alone or in combination with puerarin (P) (10, 25, and 50 *μ*M), NSCs were analyzed by Western blot for the protein expression of RagA, phospho-mTOR (p-mTOR), mTOR, phospho-p70S6K (p-p70S6K), and p70S6K. (b) Quantitation of the blots in (a). The blots in (a) were quantified by the densitometric method. The protein value was shown as means ± SD (*n* = 3). *^###^p* < 0.001 (LPS vs. control); ^∗∗∗^*p* < 0.001 (LPS+P vs. LPS). (c) Immunofluorescence staining of mTORC1 in LPS-stimulated neural stem cells. After 24 h treatment with 200 ng/ml LPS or in combination with P (10, 25, and 50 *μ*M), NSCs were incubated with mTOR and lysosomal biomarker LAMP2 antibodies, and DAPI was used to stain nuclear. The images were captured with a Zeiss fluorescence microscope (Jena, Germany). Scale bar, 20 *μ*m. (d) Quantification of mTOR and LAMP2. Colocalization of mTOR and LAMP2 was quantified by Pearson's *R* value. The results were shown as means ± SD (*n* = 3). ^∗^*p* < 0.05.

To determine the mechanisms by which RagA mediates the activity of puerarin, we focused on RagA-associated mTOR signaling pathway. For the effects of puerarin on the RagA/mTOR/p70S6K pathway, as shown in Figures 4(a) and 4(b), LPS treatment enhanced the protein expression of RagA, phospho-mTOR and phospho-p70S6K in neural stem cells. Puerarin (50 *μ*M) significantly decreased RagA expression against LPS stimulation. Moreover, puerarin (25 and 50 *μ*M) significantly decreased the expression of phospho-mTOR and phospho-p70S6K against LPS stimulation. For the effects of puerarin on the lysosomal translocation of mTORC1, as shown in Figures 4(c) and 4(d), LPS enhanced the expression of lysosomal biomarker LAMP2 and promoted the colocalization of mTOR and LAMP2 in the nucleus. Puerarin reduced LPS-induced LAMP2 expression and disturbed the colocalization of mTOR and LAMP2 in a concentration-dependent manner. Puerarin (50 *μ*M) spread the distribution of LAMP2 in the cytosol of neural stem cells against LPS stimulation.

### 3.5. Puerarin Inhibited LPS-Induced Expression of Proinflammatory Cytokines

**Figure 5:**
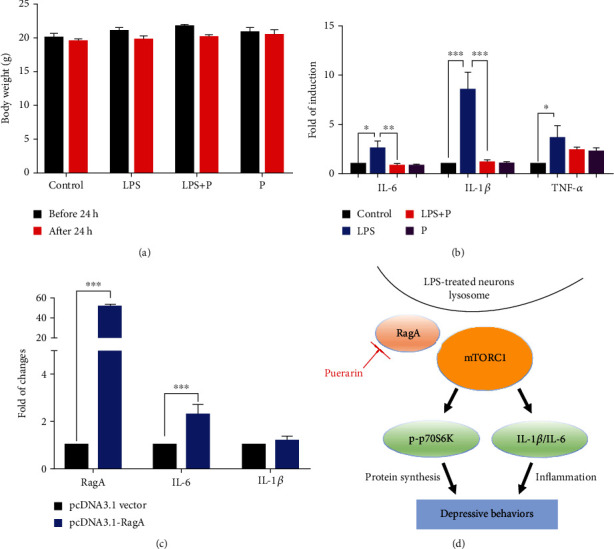
Puerarin attenuated the expression of inflammatory cytokines against LPS stimulation. (a) Body weight of experimental mice. The body weight of the mice in the indicated groups was measured before and after LPS treatment. (b) qRT-PCR quantification of representative inflammatory cytokines. The frontal cortex tissues were collected from 4 groups of mice (i.e., control, LPS, and LPS+puerarin (60 mg/kg), puerarin (60 mg/kg), *n* = 3) and analysed by qPCR analysis. The results were shown as means ± SEM (*n* = 3). ^∗^*p* < 0.05; ^∗∗^*p* < 0.01; ^∗∗∗^*p* < 0.001. (c) qRT-PCR quantification of RagA, IL-6, and IL-1*β*. Highly differentiated PC12 cells were transfected with pcDNA3.1 vector or pcDNA3.1-RagA plasmid for 24 h. The cells were collected for qPCR analysis. The data were shown as means ± SD (*n* = 3). ^∗∗∗^*p* < 0.001. (d) The diagram illustrating the potential mechanism. Puerarin targeted RagA and thereby inhibited the mTOR/p70S6K pathway and suppressed the mRNA levels of proinflammatory cytokines (e.g., IL-6 and IL-1*β*) towards the amelioration of depressive behaviors.

To study the role of RagA in the anti-inflammatory activity of puerarin, the effects of puerarin were evaluated on LPS-induced inflammatory responses in mice. Firstly, we clarified whether LPS-induced sickness in mice. After 24 h treatment, we measured the body weight of mice. As shown in Figure 5(a), LPS and puerarin did not obviously change the body weight of mice, suggesting that LPS did not cause sickness in mice. Secondly, we determined whether puerarin could suppress LPS-induced production of proinflammatory cytokines in mice. As shown in Figure 5(b), qRT-PCR analysis validated that LPS markedly induced the mRNA levels of proinflammatory genes including IL-6, IL-1*β*, and TNF-*α*. Puerarin effectively reduced the mRNA levels of IL-6 and IL-1*β* and affected TNF-*α* to a lesser extent. Thirdly, we examined the effect of ectopic RagA overexpression on cytokine synthesis. The full-length RagA cDNA was cloned in pcDNA3.1 (+) vector and subsequently introduced into highly differentiated PC12 cells. As shown in Figure 5(c), RagA transfection resulted profound increase in the expression of RagA mRNA. The mRNA level of IL-6 but not IL-1*β* was obviously elevated in RagA-transfected PC12 cells compared with that in pcDNA3.1 vector transfected cells.

## 4. Discussion

Depressive symptom may be one of the predictive signs for the onset of AD in the elderly population. Inflammation is known to increase the risk of depression in AD patients [36]. Thus, LPS was used to induce acute inflammation and depressive-like behaviors in mice [37]. Indeed, we recently found that LPS could cause the depressive-like behaviors in mice via activating RagA/mTOR/p70S6K pathway [8]. As for the therapy of depression, the conventional antidepressant drugs are challenged by gastrointestinal and sexual side effects [38, 39]. Interestingly, we recently discovered that botanical drug puerarin could effectively attenuate depression and pain in mice with SNI [23]. Based on the side-by-side comparison with the clinical drugs, puerarin and citalopram achieved similar effects on the depressive behaviors and pain in mice whereas ibuprofen appeared to be less effective. In fact, others also evaluated the antidepressant effects of puerarin in the other animal models [40]. In this study, we firstly validated that puerarin could attenuate the LPS-induced depressive-like behaviors in mice (Figures 1(c) and 1(d)). Subsequently, we focused on the molecular mechanisms underlying the antidepressant activity of puerarin. Our strategy was to identify differentially expressed genes in mice after treatment with LPS and puerarin, alone or in combination, by next-generation RNA sequencing technology. Indeed, we found that puerarin downregulated the expression of RagA mRNA by 60 folds compared with LPS-stimulated group. We further investigated the effects of puerarin on RagA/mTOR/p70S6K pathway, the accumulation of mTORC1 at the lysosomal membrane, and the production of proinflammatory cytokines.

Next-generation RNA sequencing technology is widely used to profile the transcriptomes of cultured cells and animal models for differentially expressed genes [41]. In our study, adult male mice were randomly divided into four groups: control, LPS, LPS+Puearin, and puearin. RNA sequencing profiles revealed that puerarin affected RagA and other 15 genes against LPS stimulation (Figures 2(a) and 2(b)). The functional annotation classified these genes into multicellular organism development, small ubiquitin-like modifier (SUMO) transferase activity, activation of protein kinase activity, DNA-binding transcription factor activity, protein heterodimerization activity, and other activities (Figure 2(c)). As for multicellular organism development, puerarin upregulated small subunit processome component (Utp3) and downregulated Wnt family member 7B (Wnt7b) and frizzled class receptor 1 (Fzd1). Utp3 is involved in the development of the osteosarcoma through regulating the ribosome biogenesis [42]. Wnt7b is a key ligand for the Wnt signaling pathway and mainly regulates the angiogenesis in the brain and spinal cord [43]. Fzd1 is a receptor for the Wnt signaling and regulates the adult hippocampal neurogenesis [44]. For SUMO transferase activity, puerarin upregulated the isoform Sumo2. Sumo2 is directly involved in the protein sumoylation for controlling the cognitive process in mice [45]. For the activation of protein kinase activity, puerarin upregulated insulin receptor (Insr) and calcium/calmodulin-dependent protein kinase kinase 2 (Camkk2). Insr refers to a tyrosine kinase receptor and transmitted the ligands of insulin to bind to the intracellular signaling [46]. Insr manifested as isoform A and isoform B. Insr-A was mainly expressed in the brain and uniquely expressed in the neuron. Insr-B was mainly expressed in the peripheral tissue [47]. Insr is upregulated in the entorhinal cortex and hippocampus in patients with Alzheimer's disease compared with elderly control group [48]. Camkk2 is characterized as a protein kinase for regulating cell survival and proliferation [49]. Camkk2 could decrease the expression of proinflammatory cytokines and promote LPS-treated primary microglial cells to undergo M2 polarization [50]. For protein transporter activity, puerarin upregulated adaptor-related protein complex 3 subunit sigma 2 (AP3S2). Previous study identified that some AP3S2 genetic variants are related to the onset of type-2 diabetes [51]. For DNA-binding transcription factor activity, puerarin upregulated SCAN domain containing 1 (Scand1), TATA-box-binding protein-associated factor 5 like (Taf5l), and zinc finger protein 322 (Zfp322a). Scand1 is suggested to be a potential druggable target in the treatment of anxiety disorder [52]. Scand1 is upregulated by carbon monoxide in the cortical astrocytes [53]. Taf5l is a component of the P300/CBP-associated factor (PCAF) complex with the activity to acetylate the histones [54]. Another study showed that Taf5l is an epigenetic regulator for mediating the mouse embryonic stem cell state [55]. Zfp322a regulates the pluripotency of the mouse embryonic stem cells and increases the reprogramming efficiency [56]. For protein heterodimerization activity, puerarin downregulated small GTPase RagA. RagA senses amino acids for the mTORC1 signaling pathway involving in cell growth and metabolism [14]. For other activities, puerarin downregulated cytochrome c oxidase 19 (Cox19), fat storage-inducing transmembrane protein 2 (Fitm2), and glioblastoma amplified sequence (Gbas). Cox19 is involved in the copper efflux to the mitochondria [57]. Fitm2 regulates in the normal fat storage in adipose tissues in mice and plays critical role in the cell homeostasis and the locomotor functions [58]. Gbas was expressed in the brain and heart to regulate the CREB signaling pathway, Ca^2+^ influx, and vesicular transport [59]. On the other hand, puerarin upregulated mesoderm development candidate 1 (Mesdc1) and purinergic receptor P2Y13 (P2ry13). Mesdc1 is an actin-binding protein and has an oncogenicity in the bladder cancer and hepatocellular carcinoma [60]. P2ry13 is mainly expressed in the microglia, not neuron or astrocyte. Interestingly, P2ry13 disruption enhances the proliferation of progenitor cells and the formation of new neurons [61]. Based on the gene abundance, fold changes and biological relevance, RagA was selected for further study in current study.

Our group recently revealed that LPS markedly upregulated RagA expression and activated mTORC1 pathway [8]. RagA forms heterodimers with other isoforms for different functions, for example, RagA/RagB heterodimer for binding GTP and RagC/RagD heterodimer for binding GDP [62]. Importantly, RagA senses the cellular sufficiency of amino acids for the mTOR signaling pathway [63]. When amino acids are deficient, the inactive heterodimer (RagA/B) recruits the trimeric tuberous sclerosis complex (TSC) to the lysosome, thereby preventing the association with mTORC1 activator Ras-homolog accumulated in brain (Rheb) [64]. When the amino acids are sufficient, however, heterodimer RagA/B is loaded with GTP to bind the regulatory-associated protein of mammalian target of rapamycin (Raptor), recruit mTORC1 to the lysosome surface to form complex with Rheb and activate mTORC1 [65]. The effect of mTORC1 signaling pathway is the regulation of cell growth and metabolism while dysregulation of mTORC1 is implicated in the neurodegenerative diseases, cancer, and diabetes [66]. The phosphorylation of the downstream targets including p70S6K and 4EBP1 exhibit the activity of mTORC1 [67]. Phosphorylated p70S6K promotes the protein synthesis. Phosphorylated 4EBP1 does not bind to the eukaryotic translation initiation factor 4E (EIF4E), which promotes the cap-dependent mRNA translation [68]. The p70S6K signaling pathway is a potential mechanism mediating the major depressive disorder [69]. High-fat diet caused the anxiety and anhedonia via reducing the expression of phospho-P70S6K in the frontal cortex of rats [70]. In the present study, RagA, FZD1, Fitm2, Camkk2, Ap3S2, and Mesdc1 were selected for qPCR validation. LPS increased the mRNA levels of RagA, FZD1, and Fitm2 compared with the control group. Puerarin at the dosage of 60 mg/kg significantly reduced the expression of mRNA levels of RagA, FZD1, and Fitm2 against LPS stimulation although the mRNA levels of Camkk2, Ap3S2, and Mesdc1 remained unchanged (Figure 3(a)). Puerarin effectively reduced RagA in LPS-challenged mice (Figures 3(b)–3(e)). Such results appeared to be consistent with the results of RNA-seq profiling. Neurogenesis is implicated in the depression [71]. Our previous study demonstrated that LPS activated the RagA/mTOR/p70S6K pathway and caused the lysosomal accumulation of mTORC1 in the NSCs [8]. In our study, we validated that puerarin inhibited the RagA/mTOR/p70S6K pathway and reduced the accumulation of mTORC1 lysosomal in neural stem cells (Figures 4(a)–4(d)). Moreover, LPS is well known to induce inflammation in mice [37]. In this study, we firstly confirmed that LPS and puerarin could not cause significant sickness in mice (Figure 5(a)). Such results clarified that depressive behaviors were not resulted from LPS-induced sickness. Secondly, we examined the effects of puerarin on proinflammatory cytokines. Indeed, puerarin effectively reduced corresponding mRNA level of IL-6 and IL-1*β* and affected TNF-*α* to a lesser extent in LPS-challenged mice (Figure 5(b)). As for the potential role of RagA in LPS-induced inflammatory response, we examined whether ectopic expression of RagA could influence the expression of proinflammatory cytokines (e.g., IL-6 and IL-1*β*). As result, the overexpression of RagA led to the significant increase in IL-6 mRNA levels, but not IL-1*β* mRNA in highly differentiated PC12 cells (Figure 5(c)). Interestingly, mTORC1 often cross-talks with mTORC2 to regulate the production of different cytokines [72]. Combining mTORC1/mTORC2 with pp242 or high-dose rapamycin upregulated IL-1*β* and TNF-*α* but downregulated IL-6 [73]. In addition, the mTORC1 pathway regulated IL-1*β* by dendritic cells [74]. Presumably, RagA links the mTORC1 signaling pathway to the expression of different proinflammatory cytokines in a cell context specific manner. Taken together, puerarin inhibited the RagA/mTOR/p70S6K pathway and reduced the production of proinflammatory cytokines (e.g., IL-6, IL-1*β*) against LPS stimulation. Ultimately, puerarin could effectively ameliorate LPS-induced depressive behaviors (Figure 5(d)).

## 5. Conclusions

The results from the present study not only validated that botanical drug puerarin could attenuate LPS-stimulated depressive-like behaviors in mice but also revealed that the inhibition of RagA/mTOR/p70S6K pathways could be a potential antidepressant mechanism. At the molecular level, puerarin downregulated RagA expression, reduced the lysosomal translocation of mTORC1, and inhibited the activation of mTOR/p70S6K pathway. Consequently, puerarin markedly reduced the expression of proinflammatory cytokines, especially, IL-6, in LPS-challenged mice. Thus, puerarin may be a promising antidepressant and anti-inflammatory candidate drug for the alleviation of depression in AD and other diseases.

## Data Availability

The data that support the findings of this study were available from the corresponding author upon reasonable request.

## Abbreviations

LPS: Liposaccharide
RagA: Ras-related GTP binding protein A
qRT-PCR: Quantitative real-time PCR
AD: Alzheimer's disease
IL: Interleukin
GTPases: Guanosine triphosphatases
mTOR: Mammalian target of rapamycin
GTP: Guanosine triphosphate
GDP: Guanosine diphosphate
LAMP2: Lysosome-associated membrane protein 2
p70S6K: p70 S6 kinase
4E-BP1: 4E-binding protein 1
CUMS: Chronic unpredictable mild stress
DMEM: Dulbecco's modified Eagle's medium
FBS: Fetal bovine serum
HS: Horse serum
bFGF: Basic fibroblast growth factor
EGF: Epidermal growth factor
TST: Tail suspension test
FST: Forced swim test
FC: Fold change
KEGG: Kyoto Encyclopedia of Genes and Genomes
GO: Gene Ontology
PCR: Polymerase chain reaction
NSC: Neural stem cell
PFA: Paraformaldehyde
PBS: Phosphate-buffered saline
RT: Room temperature
DAPI: 4′-6-diamidino-2-phenylindole
ANOVA: One-way analysis of variance
Utp3: Small subunit processome component
Wnt7b: Wnt family member 7B
Fzd1: Frizzled class receptor 1
Sumo2: Small ubiquitin-like modifier 2
Insr: Insulin receptor
Camkk2: Calcium/calmodulin-dependent protein kinase kinase 2
AP3S2: Adaptor-related protein complex 3 subunit sigma 2
Scand1: SCAN domain containing 1
Taf5l: TATA-box-binding protein-associated factor 5 like
Zfp322a: Zinc finger protein 322
PCAF: P300/CBP-associated factor
Cox19: Cytochrome c oxidase 19
Fitm2: Fat storage-inducing transmembrane protein 2
Gbas: Glioblastoma amplified sequence
Mesdc1: Mesoderm development candidate 1
P2ry13: Purinergic receptor P2Y13
TSC: Tuberous sclerosis complex
Rheb: Ras-homolog enriched in brain
Raptor: Regulatory-associated protein of mammalian target of rapamycin
EIF4E: Eukaryotic translation initiation factor 4E.
